# Rare pediatric synchronous bilateral testicular germ cell tumors of different pathological types: a case report

**DOI:** 10.3389/fped.2024.1339108

**Published:** 2024-01-18

**Authors:** Yikun Feng, Yu Qu, Rongde Wu, Wei Liu, Guoqiang Du

**Affiliations:** Department of Pediatric Surgery, Shandong Provincial Hospital Affiliated to Shandong First Medical University, Jinan, China

**Keywords:** BTGCTs, yolk sac tumor, mature teratoma, radical orchiectomy, testis-sparing surgery

## Abstract

The occurrence of synchronous bilateral testicular germ cell tumors (BTGCTs) of different pathologic histologic types in pediatric patients is rare. We reported a case of a left testicular yolk sac tumor (YST) combined with a right testicular mature teratoma. Left orchiectomy and right testis-sparing surgery were performed. Retroperitoneal recurrence was noted 6 months after surgery. The patient underwent reoperation for the resection of a retroperitoneal mass, which was pathologically diagnosed as a recurrent YST. A full cycle of chemotherapy was then administered. No tumor metastasis or recurrence has yet been detected. We present this new case, and we review the previous literature on synchronous BTGCTs to explore the clinicopathologic features and summarize the diagnostic and therapeutic experience. Radical orchiectomy, as the standard treatment for YSTs, should be considered with caution in patients with bilateral testicular tumors. Rapid intraoperative frozen pathology provides support for timely surgical planning. In patients with intraoperative frozen pathologic specimens suggestive of benign lesions, testis-sparing surgery is the preferred treatment option.

## Introduction

Testicular tumors in children have a low incidence, accounting for 1%–2% of pediatric solid tumors (1, 2), the most common being germ cell tumors (GCTs) (3). Testicular masses in prepubertal children are usually benign and unilateral. It is particularly rare for testicular tumors of different pathologic types to occur simultaneously on both sides. Diagnostic and therapeutic experience is largely derived from case reports. We reported a case of a synchronous bilateral testicular germ cell tumors (BTGCTs) consisting of a yolk sac tumor and a teratoma. This patient presented with a painless left-sided scrotal mass. On examination, a smaller mass was also palpable in the right scrotum. The alpha-fetoprotein level was elevated to 97,861.20 ng/ml. Postoperative pathology confirmed the diagnosis of synchronous BTGCTs of different pathologic histologic types with a yolk cystic tumor on the left and a teratoma on the right. Retroperitoneal metastases occurred 6 months after the initial surgery. The patient underwent resection of the retroperitoneal mass and is now receiving good follow-up.

## Case presentation

**Figure 1:**
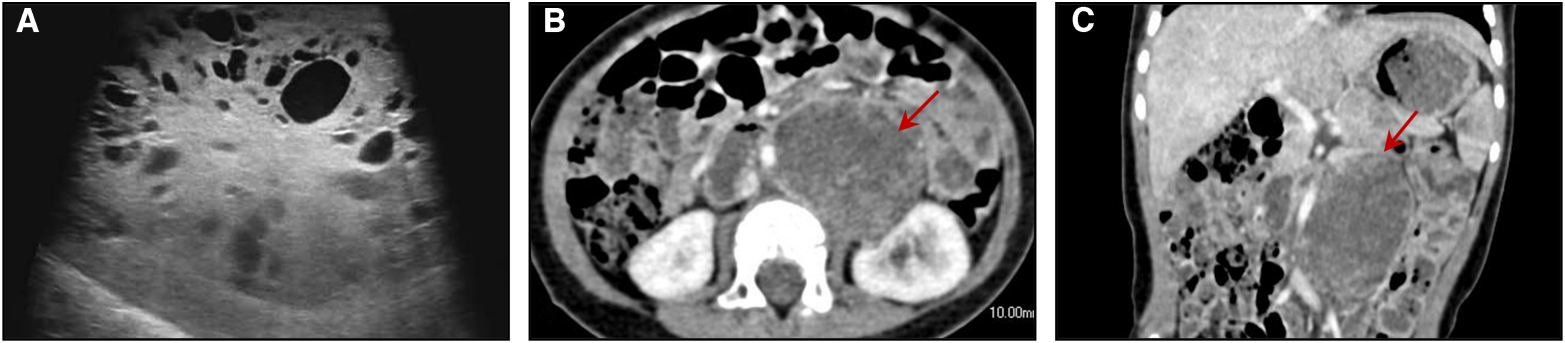
(**A**) ultrasound image showing multiple cystic structures of variable sizes in the right testis. Axial (**B**) and coronal (**C**) enhanced CT images of the abdomen show a round mass with the density of soft tissue located in the retroperitoneum of the left kidney.

A 1-year-old male child presented with a painless left scrotal mass, which had recently become enlarged and hardened with a high degree of tension. The right testis was displaced above the right scrotum and seemed to contain a cystic solid mass without obvious tenderness. Analysis of serum testicular tumor marker levels revealed that the alpha fetoprotein (AFP) concentration was 97,861.20 ng/ml (normal range: 0–7 ng/ml). Scrotal ultrasound revealed that the left testis was approximately 10.5 × 7.1 × 6.3 cm in size and had predominantly solid echoes with cystic and solid echoes in the center (Figure 1A). This patient had right cryptorchidism, and the right testis was located in the right inguinal canal, measuring approximately 1.5 × 0.7 × 0.7 cm. An inhomogeneous echogenic nodule (measuring 0.6 × 0.5 × 0.5 cm) was detected in the lower middle part of the testis; this nodule was predominantly cystic, with multiple thin septa visible. Abdominal ultrasound, retroperitoneal lymph node ultrasound and CT of the chest and abdomen showed no abnormalities.

**Figure 2:**
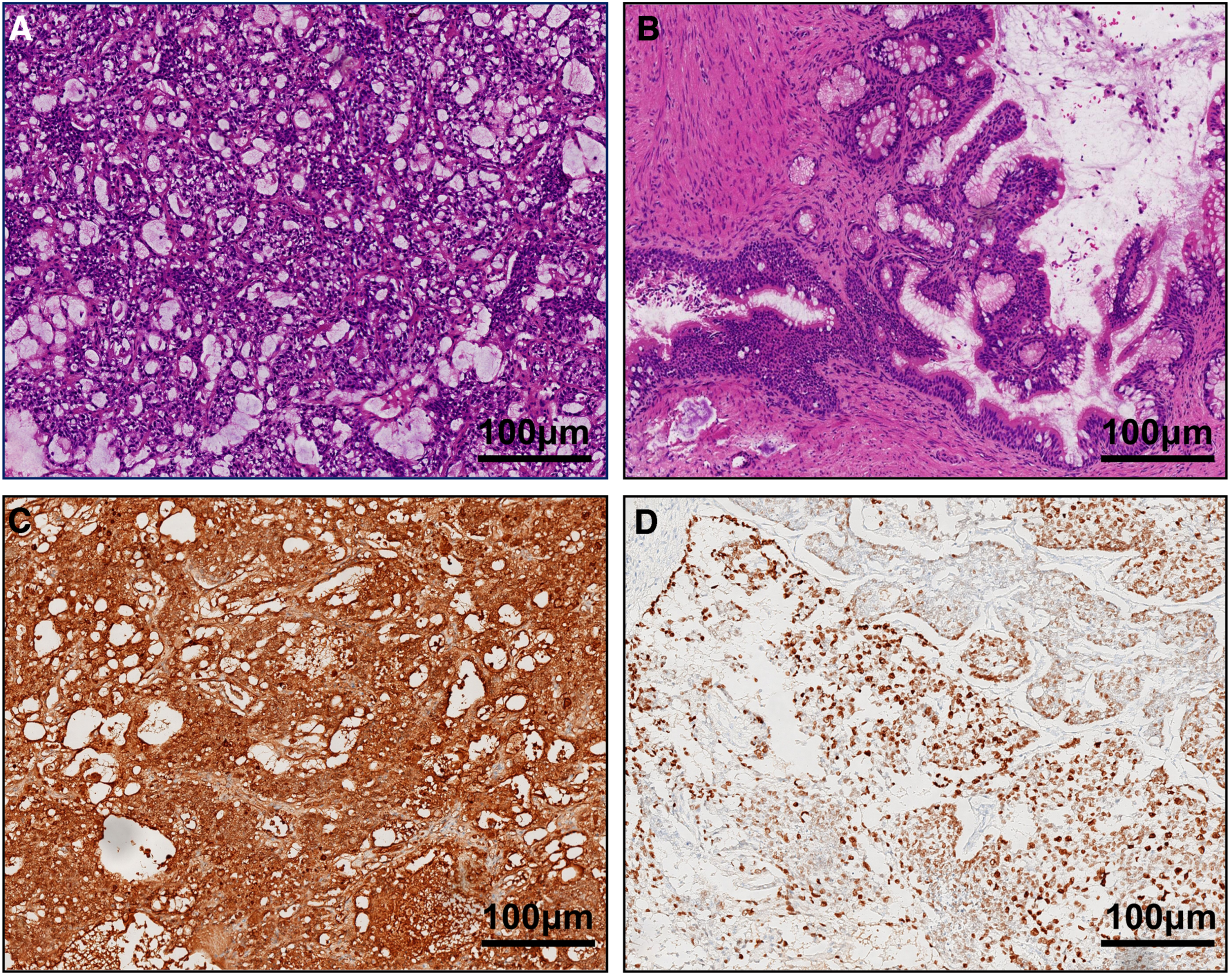
(**A**) yolk sac tumor on the left side and (**B**) mature teratoma on the right side (HE ×100). Immunohistochemical staining (×100) revealed that the tumor cells were positive for AFP (**C**) and CK (**D**).

Intraoperatively, we first blocked the left spermatic cord, and after dissecting the testicular tunica albuginea, we found that the tumor capsule was intact. The left testicular mass was removed completely, and the left testicular tumor was found to consist of a dark brown tissue with a complete capsule containing decomposed fish-like and jelly-like tissue. Rapid frozen pathology revealed a yolk sac tumor. Then, a left orchiectomy was performed. The patient underwent complete resection of the left testis and spermatic cord, with adequate hemostasis in the trauma cavity and sequential suturing of the layers. We determined preoperatively that the right testicular tumor was a benign tumor, and combined with the fact that the left testicle had undergone radical surgery, the right spermatic cord was not blocked. A cystic solid mass was observed in the parenchyma of the right testis with clear borders and a complete capsule. After complete excision of the right testicular mass along the tumor capsule, the mass was sent for examination, and rapid cryopathologic examination revealed that it was a mature cystic teratoma. The tumor parenchyma was examined, and no hemorrhages or residual tumor tissue was observed. The incision was sutured layer by layer. The patient's final pathologic diagnosis was as follows (Figure 2). We considered the 1-year-old boy to have a clinical stage I YST, so no further treatment was given. After the operation, the patient received regular follow-up, and the scrotal incision healed well without other discomfort.

However, his AFP concentration did not decrease to a normal level and was 1,750.2, 1,671.40, and 3,627.40 ng/ml at 1, 2, and 3 months postsurgery, respectively. Abdominal enhanced CT showed a round mass with the density of soft tissue (measuring 4.5 × 3.3 cm) located in the retroperitoneum of the left kidney, and multiple small lymph nodes were observed in the abdominal, retroperitoneal, pelvic and bilateral inguinal regions (Figures 1B,C). A retroperitoneal tumor was considered after careful analysis by the doctors. A second resection was performed, and the pathological findings revealed that the yolk sac tumor had undergone retroperitoneal metastasis. Seven cycles of chemotherapy were administered after the operation. At present, chemotherapy has ended, and his AFP level has returned to normal. To date, the child has been followed up for 2 years, and no tumor metastasis or recurrence has been detected.

## Discussion

Research has clearly demonstrated fundamental differences in incidence and histology between testicular tumors in childhood and those in adolescence and adulthood (4, 5). Teratomas are usually benign in prepubertal children and account for the largest proportion of intratesticular tumors, approximately 40% (6). Yolk sac tumors are the main prepubertal malignant germ cell tumors and may account for approximately 15% of prepubertal tumors in boys. The incidence of prepubertal testicular tumors is low, and the occurrence of synchronous BTGCTs of different pathologic histologic types is exceedingly rare. Table 1 shows 5 previous cases of synchronous BTGCTs of different pathological types (7–11). Mature teratomas and YSTs are the most common benign and malignant testicular tumors in the pediatric population (12), and they are also common pairs of bilateral testicular tumors that have been reported previously. Synchronous BTGCTs of different pathological types present some challenges in terms of diagnosis and treatment.

**Table 1:** Published studies on synchronous BTGCTs of different pathological types in children.

Authors, date	Age (month)	Pathology result	Treatment
Royal et al., 1994 (7)	24 months	R: MGCT (95% YST) L: Teratoma	Bilateral orchiectomy
Luo et al., 1998 (8)	7 months	R: Teratoma L: YST	Bilateral orchiectomy
Dong et al., 2017 (9)	7 months	R: Teratoma L: YST	R: TSS L: Orchiectomy
Li et al., 2019 (10)	19 months	R: Teratoma L: YST	R: TSS L: Orchiectomy
Kebudi et al., 2019 (11)	23 months	R: YST L: Teratoma	R: Orchiectomy L: TSS
Present case, 2023	12 months	R: Teratoma L: YST	R: TSS L: Orchiectomy

MGCT, mixed germ cell tumor; YST, yolk sac tumor; TSS, testis-sparing surgery.

Testicular tumors are mostly detected by parents or children when abnormalities in the testicles are observed. Most of the parents reported that their children's scrotum was enlarged or that they palpated testicles, found them to have an abnormal texture and came to the hospital for consultation. A small number of children come to the hospital for treatment of other diseases, or testicular tumors are occasionally discovered during routine physical examinations. Ultrasound is preferred for the diagnosis of testicular tumors, with a sensitivity of up to 100% (13), but it does not completely enable doctors to distinguish between benign and malignant tumors. The combination of these parameters with the alpha-fetoprotein test can play a role in accurately evaluating these tumors. When elevated AFP is present in a child older than 1 year with a testicular tumor, the tumor may be considered to have a yolk sac component (14). The ultrasound presentation of testicular teratomas mainly reveals a mixed cystic-solid echogenic mass, some of which are strongly echogenic, and color Doppler findings reveal the presence of punctate blood flow signals in the parenchymal part of the mass. On ultrasound, yolk cystic tumors are mainly hypoechoic masses with blurred borders, multiple clusters of strong echoes may be present inside, and color Doppler examination findings reveal that blood flow signals within the masses are relatively abundant. In patients with yolk sac tumors, staging should be performed, including MRI of the abdomen and CT of the chest. If there is any suspicion of a tumor that is not confined to an organ, the patient should be referred to a pediatric oncologist (15).

Current experience in the treatment of bilateral testicular tumors is detailed in previous case reports. The serum AFP concentration and scrotal ultrasound combined with frozen-section examination help to precisely identify benign and malignant tumors preoperatively or intraoperatively and play important roles in deciding upon treatment strategies (16). For bilateral testicular tumors with YSTs, treatment of the malignant side should be in accordance with the standard management of unilateral testicular YSTs. In terms of YSTs, 80% are stage I. Preoperative ultrasound with a strong suspicion of YST and abnormally elevated AFP can lead directly to radical orchiectomy. If it is not possible to identify before surgery, the spermatic cord can be blocked at the internal ring, and the mass can be excised and sent for frozen pathological examination. The choice of radical orchiectomy or TSS is then determined by the results of the frozen pathological analysis. Retroperitoneal lymph node dissection (RPLND) is not routinely recommended for testicular YSTs in children and is recommended only for patients with residual retroperitoneal masses or for patients with persistently elevated AFP levels following chemotherapy or orchiectomy (17). In this report, given that the patient had bilateral testicular tumors, our diagnostic and therapeutic challenge was to determine the best surgical approach. On the one hand, it is important to ensure that the tumor is removed safely and completely. On the other hand, it is also important to weigh the need to preserve as much as possible of one side of the testis to support the patient's future physical and psychological needs. After careful preoperative evaluation of the patient, it was initially determined that the left testicular tumor was of malignant origin, while the right side was benign with no distant metastasis. Intraoperatively, the left spermatic cord was blocked to prevent hematogenous dissemination. After complete excision of the left testicular mass and rapid cryopathologic examination to confirm the diagnosis of YST, the left testis and spermatic cord were removed. In this case, preservation of the right testis was important. Considering the preoperative ultrasound diagnosis of a mature teratoma on the right side, the right spermatic cord was not blocked. The right testicular tumor was excised thoroughly and successfully. Fortunately, the right testicle could be preserved because pathology revealed a mature cystic teratoma.

The patient in the present case presented with retroperitoneal recurrence at 6 months after the initial surgical procedure, and reoperation and chemotherapy were effective. The Children's Oncology Group (COG) recommends surveillance for all patients with stage I disease after radical surgery (3). Long-term postoperative surveillance of the serum AFP concentration and ultrasound examinations are necessary. The serum AFP level in patients with testicular yolk sac tumors who have higher-than-normal AFP levels before surgery should return to normal approximately 4–6 weeks after surgery. If tumor markers are continuously measured after surgery and AFP levels continue not to decrease or decrease and then increase 4–6 weeks after surgery, tumor remnants or metastasis should be suspected, and further combined adjuvant chemotherapy should be administered (16). All prepubertal testicular teratomas are benign, whether mature or immature (18). TSS is sufficient and does not require further treatment or follow-up, which is advantageous for ensuring the long-term prognosis of benign prepubertal testicular GCTs, maintaining hormonal function and fertility (19) and reducing the psychological impact on prepubertal patients. Therefore, it is more important to choose surgical procedures carefully on an individual basis in a prepubertal population with BTGCTs.

The prognosis for YSTs in children is good, with survival rates approaching 100% for all stages. A detailed follow-up plan should be developed for children with YSTs. The AFP concentration should be tested every month for 2 years after surgery and chest x-ray and/or CT of the lungs, abdominal and/or pelvic ultrasound, and abdominal and/or pelvic CT should be performed every 3 months in the first year and every 3–6 months in the second year after surgery (20).

## Conclusion

Synchronous primary BTGCTs with teratomas and YSTs are extremely rare. Radical orchiectomy is the standard treatment for YSTs. In patients with intraoperative frozen pathologic specimens suggestive of benign lesions, testis-sparing surgery is the preferred treatment option.

## Data Availability

The original contributions presented in the study are included in the article/Supplementary Material, further inquiries can be directed to the corresponding author.
